# Potential of used frying oil in paving material: solution to environmental pollution problem

**DOI:** 10.1007/s11356-017-8793-z

**Published:** 2017-03-28

**Authors:** Dimple Singh-Ackbarali, Rean Maharaj, Nazim Mohamed, Vitra Ramjattan-Harry

**Affiliations:** The University of Trinidad and Tobago, O’Meara Industrial Park, 74-98 Churchill Roosevelt Highway, Arima, Trinidad and Tobago

**Keywords:** Used frying oil, Trinidad Lake Asphalt, Trinidad Petroleum Bitumen, Rutting resistance, Fatigue cracking

## Abstract

The improper disposal of used frying oil (UFO) presents numerous ecological, environmental and municipal problems. Of great concern is the resultant blockage of municipal drainage systems and water treatment facilities, harm to wildlife when they become coated in it and detriment to aquatic life and ecosystems due to the depletion of the oxygen content in water bodies such as rivers and lakes that have become contaminated. Statistics show that in Trinidad and Tobago, in excess of one million liters of used cooking oil is collected annually from various restaurant chains. This paper investigated the potential of using UFO as a performance enhancing additive for road paving applications utilizing Trinidad Lake Asphalt (TLA) and Trinidad Petroleum Bitumen (TPB) as a mitigation strategy for improper UFO disposal. Modified blends containing various additions of UFO (2–10% wt) were prepared for the TLA and TPB asphaltic binders. Results demonstrated in terms of stiffness, increasing the dosage of UFO in TLA and TPB base binders resulted in a gradual decrease in stiffness (G* value decreased). In terms of elasticity, increasing the dosage of the UFO additive in TLA resulted in a general decrease in the elasticity of the blends indicated by an increase in phase angle or phase lag (*δ*). Increasing dosages of the UFO additive in TPB resulted in a significant decrease in δ where the most elastic blend was at the 6% UFO level. TLA and UFO-TLA modified blends exhibited significantly lower values of δ and higher values of G* confirming the superiority of the TLA material. Incorporation of the UFO in the blends led to a decrease in the rutting resistance and increase in the fatigue cracking resistance (decrease in G*/sin*δ* and G*sin*δ*, respectively). This study highlighted the potential for the reuse of UFO as an asphalt modifier capable of producing customized UFO modified asphaltic blends for special applications and confirms its feasibility as an environmentally attractive means of reusing the waste/hazardous UFO material locally.

## Introduction

### The environmental problem

Frying oil is vegetable and animal oil that is used to fry food at high temperatures by the food industry, restaurants/food service establishments, and homes. The used frying oil (UFO) generated by these activities has become a major environmental and ecological issue, especially since it is usually indiscriminately discarded after use, into municipal landfills or poured down drains without any treatment (Patil et al. 2012, Zhang et al. 2012, Dias et al. 2014).

Petroleum oils, vegetable oils, and animal fats share common physical properties and produce similar environmental effects as outlined by literature (EPA 2015, Rodewald 2015). They include:

- Suffocation of animals and plants that have been coated with oil
- Eutrophication due to micro-organisms, phytoplankton, and algae which use the UFO as a food source
- Reduction in dissolved oxygen content and the death of aquatic plants and animals as a result of the existence of the layer of oil on water bodies which prevents sunlight from getting to aquatic plants stifling photosynthesis
- Rancid odor production
- Clogging of drainage systems and water treatment plants
- Isolation of soil from air and water, killing the earth worms, and bacteria necessary for regeneration of plants
- Proliferation of rats and vermin that feed on the solidified waste cooking oil which creates a pest control problem or health hazard.

In Trinidad and Tobago, the amended Water Pollution Rules (WASA 2006) targets commercial business activities using cooking oil such as restaurants, food service companies, and even households where these entities are required by law to register with the Environmental Management Authority (EMA). However according to an interview with a managing director of a company that collects and recycles used cooking oil, the manager stated that the laws on cooking oil disposal is not enforced as it is either frozen and thrown away or poured down the drain.

Information collected in 2010 by the Trinidad and Tobago Central Statistical Office reported that there were 317 food and drink processing establishments and 297 hotel and guest establishments in the country. A study found that a popular internationally based fried chicken fast food outlet used approximately 151.4 L of oil per week and when this is translated to all of its 52 outlets; over 409,000 L of UFO can be collected annually from this one franchise alone (John and Seetahal 2008). It is estimated that commercial establishments would use 30% of the available edible oil, 55,315 L of edible oil a day (Wyse-Mason and Beckles 2012). The remaining 70% of the consumed edible oil are utilized by residential households that are not required to have disposal facilities such as oil separators, grease traps, waste water sumps, or have their used cooking oil collected by recycling or treatment and disposal companies. This allows a significant quantity of UFO to be disposed of down the sink and drain, onto the ground, and into the garbage. Currently, the UFO generated commercially from Trinidad restaurants is contracted to one company who indicated that they collect up to one million liters of UFO annually.

### Possible solutions

Used oil is the “single largest environmentally hazardous recyclable material” (MARRC 2001) and a spill of used oil as small as 1 L can potentially contaminate a million liters of fresh water. The recycling of waste oil is becoming a viable alternative in mitigating the associated environmental and ecological problems (El-Fadel and Khoury 2001). However, developing countries struggle to properly manage their used oil due to inadequate collection services and limited utilization of recycling. Third world countries are lagging behind in this regard as they have low awareness regarding recycling of waste materials, not yet developed effective legislation, and have not yet selected lead agencies responsible for rules, regulations, and enforcement legislation (Batayne et al. 2008, Kahn et al. 2009). Progress is being made in this region, however, as the Basel Convention Regional Centre for Training and Technology Transfer for the Caribbean Region (BCRC-Caribbean) has a new central focus which shifts away from the strict prohibition of the movement of hazardous wastes from one party to another, towards the recognition of waste as a resource which can stimulate economic development and create new employment opportunities, more so among civil society groups and small business entrepreneurs. This new focus encourages waste prevention and minimization at source and waste recovery, reuse, and recycling as downstream value added components of the waste stream (Basel Convention Region Business Plan 2012). In order for a reduction in negative impacts of improper disposal of UFO in Trinidad and Tobago the following must be done:

- Update national inventory of use and disposal
- Review policy and enable legislation to facilitate waste oil collection, re-refining, disposal and destruction.
- Conduct strategic assessment of appropriate technologies that can be applied
- Develop pilot project with private sector investors
- Review fuel subsidies in Trinidad and Tobago so that local market will be inviting to the use of alternative fuel.

Many researchers have studied the potential use of recycled UFO by integration into the food chain through animal feeds, production of soaps, or conversion to biodiesel; however, limited information exist on its use of UFO as an additive in asphalt pavement binders (Bronislaw 2014, Deba et al. 2015, Panadare and Rathod 2015).

### Challenges with past solutions

Integrating UFO into the food chain through animal feed, can be a potential cause of human health problems as there is some evidence that highly oxidized fats formed during frying where oils are exposed to high temperatures in the presence of atmospheric oxygen, may have carcinogenic properties (Chang and Peterson 1978, Azpilicueta and Remirez 1991, Costa Neto et al. 2000, Panadare and Rathod 2015). The use of waste or used fats and oils in animal feedstock as an additive can also be problematic as when it becomes rancid, it imparts an objectionable odor and decreases palatability of the feed. Additionally, when excess fat or oil exceeds 6% of the feeds dry matter, inhibition of fiber digestion in rumens can occur (Engstrom et al. 1994, Panadare and Rathod 2015). Digestive disturbances, diarrhea, and reduced feed intake may occur if excessive levels of fat are fed to animals. There are several positives when looking at UFO as a fuel source for biodiesel (Sunde et al. 2011, Thamsiriroj and Murphy 2011, Bronislaw 2014) however, before the UFO can be used in saponification and biodiesel production, investments have to be made to pre-treat the waste material via filtration and esterification to remove any free fatty acids (Chang and Peterson 1978, Bronislaw 2014). While pre-treatment for the UFO to be converted to biodiesel may not be expensive, the cost of converting a diesel engine to run on UFO can cost up to TT$15000.00 (Trinidad and Tobago Newsday 2010), which may be a deterrent for citizens/individuals.

### Background and new proposed solution

Asphalts and bitumen are both used together with mineral aggregates to construct roads/pavements. The performance of these road pavements depend on the properties of the asphalt and the bitumen which are the only deformable components in the mixture. Both these systems have thermal susceptibilities and can become deformed due to weathering, moisture damage, heavy traffic, or embrittlement due to the chemical oxidation of functional groups within the asphalt. These limitations can be overcome as their performance characteristics significantly modified by modification with polymeric materials (Zhu et al. 2014, Maynard et al. 2015). It has been reported that polymer modified asphalt can increase the shelf life of pavements by up to 10 years (Dwyer and Betts 2011, Boyer 2013). The blending of recycled asphalt with UFO has been shown to improve the performance qualities of the resulting blends as the fatty acids present in UFO has been shown to act as cohesive agent, reducing the high viscosity of the aged, recycled binders, facilitating homogenous mixing and reducing surface tension of the aggregate and coated binder, when integrated with new pavement materials (Huh 2012). Other past evaluations of binder performance (Asli et al. 2012, Zargar et al. 2012) showed that a 3–4% by weight addition of the UFO gave similar viscosity results compared to the original bitumen material. It was also reported that the used of vegetable oil decreases the stiffness of the aging mixture (Bailey and Philips 2010).

Despite the existence of studies using other asphaltic binders from other sources, the influence of polymeric additives on the rheological properties of Trinidad asphaltic materials cannot be generalized and must be independently investigated as a clear relationship between the differences in the quality of asphalt (different compositions) from different sources and the resulting performance qualities of the binders exist; asphaltic materials with the same specifications can often produce pavements of varying physical properties, performance, and serviceability (Oyenkunle 2006, Oyenkunle 2007, Mohamed et al. 2016). TLA is an asphaltic material of unique composition containing significantly higher asphaltene content compared to other refinery bitumen such as TPB. TLA contains kaolinitic clay not present in TPB and other refinery bitumen. These compositional differences have been shown to influence the flow, colloidal characteristics, and rheological properties of asphaltic systems which ultimately influences their performance attributes. A literature survey shows that previous studies measuring the influence of UFO on the rheological properties of the asphaltic materials TLA and TPB, indigenous to Trinidad and Tobago have proven to be limited.

Despite the reported enhancement of asphaltic materials modified with polymeric additives, there are some associated difficulties. Polymer modified asphaltic materials have been linked to increased amounts of polycyclic aromatic hydrocarbons (PAHs) being leached into storm water and contaminating water bodies. PAHs consist of over a hundred organic compounds with two or more aromatic rings that occur together as mixtures. They can be concentrated by incomplete burning of carbon-containing material; sources include tyres and crumbling asphalt. Road pavement material and car park sealants can contribute significant amounts of PAHs to water ways via storm water which can be toxic to aquatic animals (Wright et al. 2009). A 2006 evaluation of PAHS in frying oils found that both before and after frying, the benzo-a-pyrene concentration in edible oils ranged from trace to 0.7 ppb, well below the 2 ppb limit for PAHs in foods recently proposed by the European Community (Purcaro et al. 2006). Research showed that crumb rubber samples analyzed had high levels of PAHs and Zinc (Marsili et al. 2015).

This paper seeks to fill the gap of research investigating the influence of UFO on the rheological properties of TLA and TPB asphaltic materials indigenous to Trinidad and Tobago, and hence assess its potential as an environmentally attractive means of reusing the waste/hazardous UFO material locally.

## Experimental

### Materials sources

A gallon of used frying oil (UFO) was obtained from a commercial restaurant in South Trinidad. Trinidad Lake Asphalt (TLA) and Trinidad Petroleum Bitumen (TPB) were sourced from the Lake Asphalt Company of Trinidad and Tobago and the Petroleum Company of Trinidad and Tobago Limited, respectively.

### Sample preparation

Aluminum cans of approximately 500 cm^3^ were filled with 250–260 g of asphalt and put in a thermoelectric heater Thermo Scientific Precision (Model 6555) where the temperature was raised to 200 °C. A digital IKA (Model RW20D) high shear mixer was then immersed in the can and set to 3000 rpm. The UFO was added gradually while the system was kept at a temperature of 200 ± 1 °C. Each TLA-UFO and TPB-UFO blend was formed from 0, 2, 4, 6, 8, and 10% of UFO by weight. At the end of mixing, each blend was split into different cans, transferred to a desiccator and stored under static conditions and in an oxygen-free environment. After 24 h period of curing, the cans were taken out, remixed using high shear mixer, and the molten mixtures were then cast into a ring stamp with 25 mm diameter and 1 mm thickness for subsequent rheological testing. Before testing, the samples were cooled at room temperature and stored in a Fisher Isotemp freezer at −20 °C.

### Rheological measurements

The rheological characterization of the various asphalt blends were studied using an oscillatory dynamic shear rheometer (ATS RheoSystems) operated within the linear domain under strain control. The test geometries were plate to plate (diameters 25 and 1 mm gap). Viscosity measurements were conducted in the temperature range 40–90 °C and frequency range was 0.1–15.91 Hz. The analysis was performed under the strain control mode and the complex modulus (G*) and phase angle (*δ*) values at the different oscillating frequencies and temperatures were calculated using the instruments software.

## Results and discussion

The use of measurements using dynamic shear rheometer (DSR) rheological properties of Trinidad asphalt materials at temperatures from high to intermediate values are an important consideration in understanding pavement distress characteristics such as pavement deformation due to rutting and shearing. The understanding and application of this technique is well-documented by Kim (2009) and has been successfully utilized for the rheological characterization of polymer modified asphaltic materials including the measurement of key performance attributes of fatigue cracking and rutting resistance (Hosein et al. 2013, Maharaj and Maharaj 2015, Maynard et al. 2015).

Deformation in asphalt material consists of three types:

- Instant elastic recoverable strain
- Delayed elastic recoverable strain
- Permanent non-recoverable strain (or viscous flow)

Most critical among these is the permanent non-recoverable strain or viscous flow parameter which determines the permanent deformation of the traffic asphalt pavement due to repeated loading forces. Table 1 below describes the different parameters that were tested with the DSR, and introduces the characteristics of the material that can be interpreted when analyzing the results.

**Table 1:** The different parameters and characteristics that were tested and analyzed using the DSR

Parameter	Characteristics that can be interpreted
Complex shear modulus, G*	Represents the total resistance of the asphalt/bitumen sample to deformation (or stiffness) caused by repeated pulses of small angle oscillations by the plates of the DSR, high values are desirable for a stiffer material low values are associated with a softer material
Phase angle or the phase lag, *δ*	Represents the degree of the elasticity of the material, high values are associated with high viscosity materials, low values are associated with highly elastic materials resistance
Rutting resistance parameter G*/sin*δ*	Higher values of G*/sin*δ* will result in higher rutting resistance of material, lower values of G*/sin*δ* will result in lower rutting resistance of the material
Fatigue cracking parameter G*sin*δ*	Lower values of G*sin*δ* will result in higher fatigue cracking resistance of material higher values of G*in lower fatigue cracking resistance of the material

**Fig. 1:**
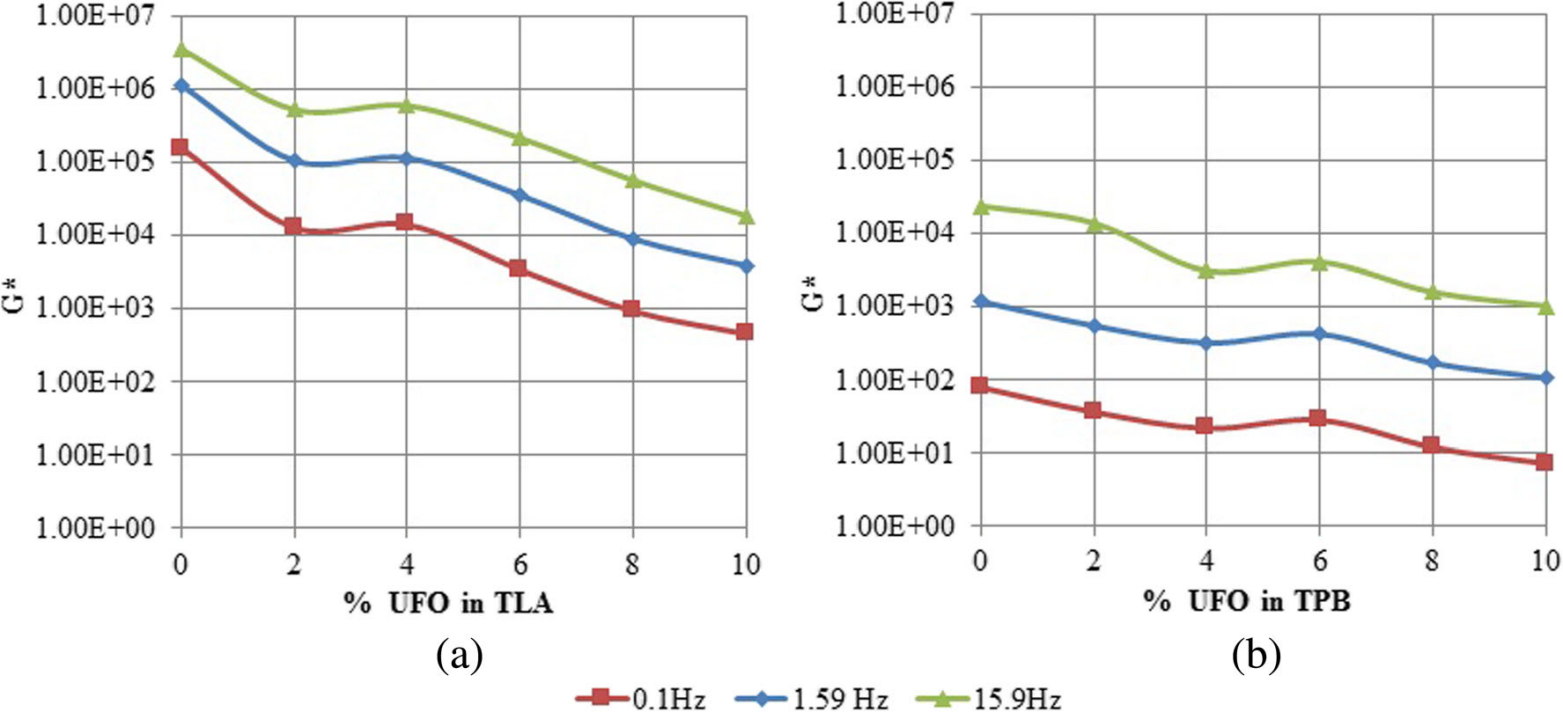
The variation of G* with increasing concentration of UFO for TLA and TPB binders at various oscillating load frequencies and at 60 °C

Figure 1a, b show the changes in complex shear modulus (G*) at various oscillating load frequencies at 60 °C, as the concentration of the added UFO was increased for TLA and TPB binders, respectively.

A comparison of Fig. 1a, b shows that unmodified TLA and the UFO-TLA blends exhibited higher G* values than TPB and UFO-TPB blends indicating that they are generally stiffer. This observation was consistent with the findings of previous researchers (Hosein et al. 2013, Maharaj et al. 2014, Maynard et al. 2015). The results show that for both the TLA and TPB base binders, as the concentration of the added UFO was increased gradually, the stiffness generally decreased (G* value decreased). A similar observation was recorded by Raghavan and Kaler (2001), Borhan et al. (2009), and Singh-Ackbarali and Maharaj (2011), and it has been suggested that the decrease in G* observed can be attributed to an increased solvency of the maltenes present in the asphaltic materials in the fatty acids present in the UFO; softening the intermolecular cross-linkages which resulted in the modified blends having reduced ability to withstand elongation. When aggregate is added to these UFO modified asphaltic blends, it is expected that the mechanical properties of the pavement would be improved as the reduced viscosity will result in a reduction in the surface tension between the aggregate and the binder coating, expelling trapped air and increasing interfacial cohesion between the asphaltic binder and aggregate.

**Fig. 2:**
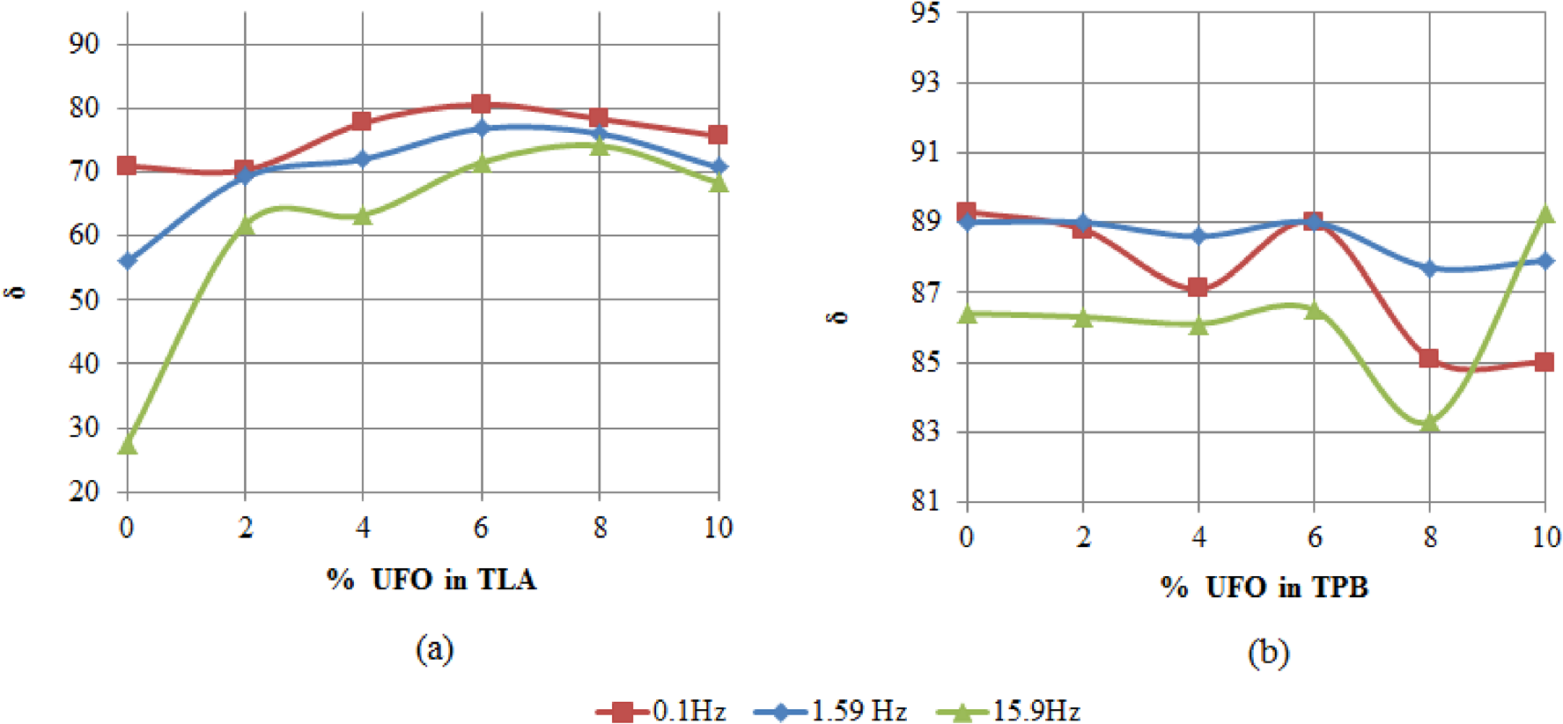
a, b The variation of *δ* with increasing concentration of UFO for TLA and TPB binders at various oscillating load frequencies and at 60 °C

Figures 2a, b show the variation of the phase angle *δ* with increasing concentration of UFO for TLA and TPB, respectively at various oscillating load frequencies and at 60 °C.

The results demonstrated that *δ* was generally higher for the TPB and UFO-TPB blends indicating that these blends had lower elasticity. The observation that *δ* was almost 90° for unmodified TPB indicated that the material behaved almost like a viscous liquid. On the other hand, the TLA and UFO-TLA blends had significantly lower values of *δ* indicating that these blends were significantly more elastic. The relatively higher values of G* and lower values of *δ* observed for the TLA based binder (relatively stiffer and more elastic) offer supporting rheological evidence confirming TLA’s world renowned superior qualities and its consequential use as an additive to improve the properties of other refinery bitumen including TLA (Widyatmoko and Elliott 2008). The effect of increasing the concentration of the UFO additive in TLA resulted in a general decrease in the elasticity of the blends indicated by an increase in *δ*. The effect of increasing the concentration of the UFO additive in TPB resulted in a significant decrease in *δ* at the 6% UFO level indicating a superior elastic UFO blend.

**Fig. 3:**
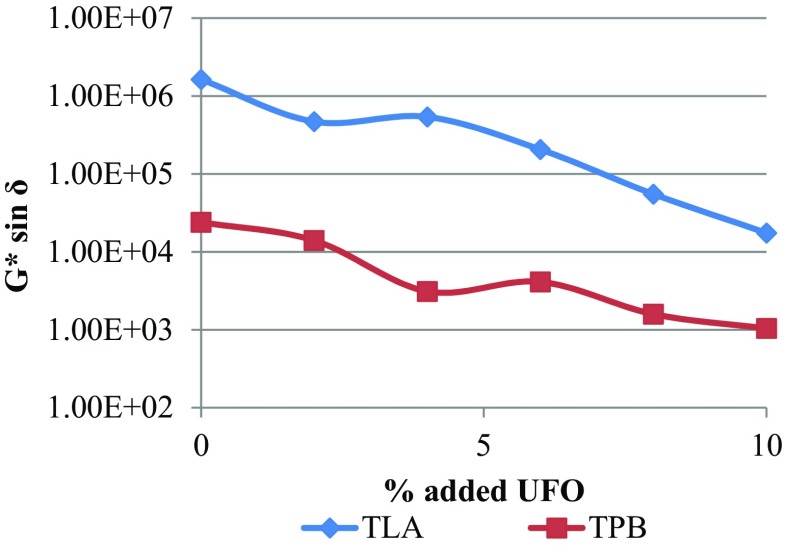
The variation of the fatigue cracking resistance parameter (G*sin*δ*) with increasing concentration of UFO in TLA and TPB mix at various oscillating frequencies at 60 °C

The variation of the fatigue cracking resistance parameter (G*sin*δ*) with increasing concentration of UFO in TLA and TPB at various oscillating frequencies at 60 °C is shown in Fig. 3.

**Fig. 4:**
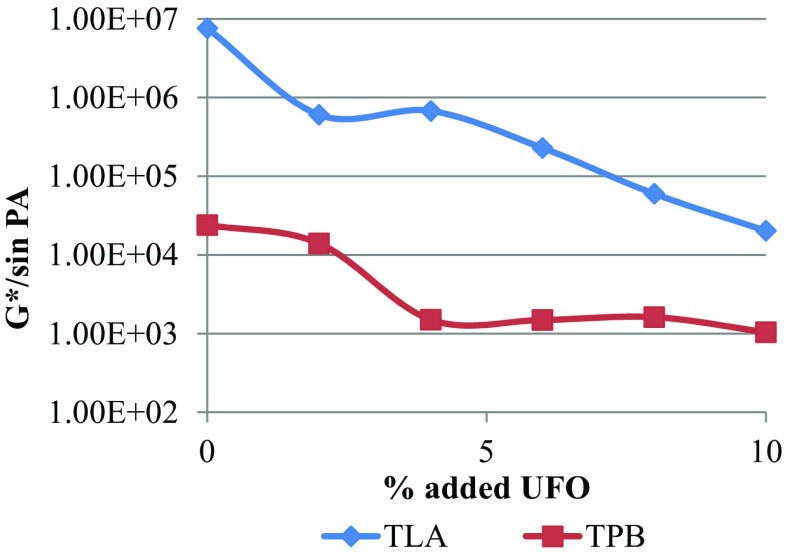
The variation of the rutting resistance parameter (G*/sin*δ*) with increasing concentration of UFO in TLA and TPB blends at three oscillating frequencies

The trend of decreasing G*sin*δ* values as the % UFO was increased for both TLA and TPB indicates higher fatigue cracking resistance as the UFO component was increased. The TLA and the UFO-TLA blends exhibited higher G*sin*δ* values (lower fatigue cracking resistance values) compared to TPB and UFO-TPB blends. The variation of the rutting resistance parameter (G*/sin*δ*) with increasing concentration of UFO in TLA and TPB blends at three oscillating frequencies, respectively are shown in Fig. 4.

The results clearly indicate that the addition of UFO in the TLA and TPB binders resulted in a decrease in the rutting resistance (a decrease in G*/sin*δ* values) of the resulting blends. The unmodified TLA and TPB exhibited superior rutting resistance properties compared to the UFO modified blends. The superiority of the TLA and its UFO-TLA blends was again evident as the G*/sin*δ* (rutting resistance) values were significantly higher than TPB and the UFO-TPB blends.

Differences between the rheological properties observed for the TLA and TPB blends offer supporting evidence for previous studies where it has been shown that the influence of additives on the rheological properties of asphaltic materials from different sources cannot be generalized and must be independently investigated as there exists a clear proven relationship between the differences in the quality of asphalt (different compositions) from different sources and the resulting performance qualities of the binders exist; asphaltic materials with the same specifications can often produce pavements of varying physical properties, performance, and serviceability (Mohamed et al. 2016, 28).

## Conclusion

The rheological analysis of modified TLA and TPB blends containing various additions of UFO (2–10% wt) demonstrated the following:

- In terms of stiffness, increasing the concentration of UFO in the TLA and TPB base binders resulted in a corresponding decrease in stiffness (G* value decreased).
- In terms of elasticity, increasing the concentration of the UFO additive in TLA resulted in a general decrease in the elasticity of the blends indicated by an increase in *δ*. Increasing the concentration of the UFO additive in TPB resulted in a significant decrease in *δ* where the most elastic blend was at the 6% UFO level.
- TLA and UFO-TLA modified blends exhibited significantly lower values of *δ* and higher values of G* confirming the superiority of the TLA material.
- Incorporation of the UFO in the blends led to a decrease in the rutting resistance and increase in the fatigue cracking resistance (decrease in G*/sin*δ* and G*sin*δ*, respectively).

This study demonstrated the potential reuse of UFO as an asphalt modifier capable of producing customized UFO modified asphaltic blends for special applications. It also demonstrates the feasibility of the reuse strategy as an environmentally attractive means of disposal of the waste/hazardous UFO material locally.
